# Penetrating Bladder Trauma: A High Risk Factor for Associated Rectal Injury

**DOI:** 10.1155/2014/386280

**Published:** 2014-01-09

**Authors:** B. M. Pereira, L. O. Reis, T. R. Calderan, C. C. de Campos, G. P. Fraga

**Affiliations:** ^1^Division of Trauma Surgery, Faculty of Medical Sciences, University of Campinas (UNICAMP), 13083-887 Campinas, SP, Brazil; ^2^Division of Urology, Faculty of Medical Sciences, University of Campinas (UNICAMP), Rua Tessália Vieira de Camargo 126, Cidade Universitária “Zeferino Vaz,” 13083-887 Campinas, SP, Brazil; ^3^Faculty of Medicine, Pontifical Catholic University of Campinas (PUC), 13083-887 Campinas, SP, Brazil; ^4^Department of Surgery, Faculty of Medical Sciences, University of Campinas (UNICAMP), 13083-887 Campinas, SP, Brazil

## Abstract

Demographics and mechanisms were analyzed in prospectively maintained level one trauma center database 1990–2012. Among 2,693 trauma laparotomies, 113 (4.1%) presented bladder lesions; 51.3% with penetrating injuries (*n* = 58); 41.3% (*n* = 24) with rectal injuries, males corresponding to 95.8%, mean age 29.8 years; 79.1% with gunshot wounds and 20.9% with impalement; 91.6% arriving the emergence room awake (Glasgow 14-15), hemodynamically stable (average systolic blood pressure 119.5 mmHg); 95.8% with macroscopic hematuria; and 100% with penetrating stigmata. Physical exam was not sensitive for rectal injuries, showing only 25% positivity in patients. While 60% of intraperitoneal bladder injuries were surgically repaired, extraperitoneal ones were mainly repaired using Foley catheter alone (87.6%). Rectal injuries, intraperitoneal in 66.6% of the cases and AAST-OIS grade II in 45.8%, were treated with primary suture plus protective colostomy; 8.3% were sigmoid injuries, and 70.8% of all injuries had a minimum stool spillage. Mean injury severity score was 19; mean length of stay 10 days; 20% of complications with no death. Concomitant rectal injuries were not a determinant prognosis factor. Penetrating bladder injuries are highly associated with rectal injuries (41.3%). Heme-negative rectal examination should not preclude proctoscopy and eventually rectal surgical exploration (only 25% sensitivity).

## 1. Introduction

Penetrating trauma implies that either a gunshot wound or a stab wound has entered the abdominal cavity. The gunshot wound is associated with high-energy transfer and the extent of intra-abdominal injuries is difficult to predict. Both the path of the missile and secondary missiles are unpredictable, as well as bone fragments or fragments of the bullet that can inflict other injuries.

The velocity of assault rifles and hunting firearms is much higher than that of civilian handguns and therefore has a much higher energy transfer to the tissue. Stab wound injuries can be inflicted by many objects other than knives, including knitting needles, garden forks, fence railing, wire, pencils, and pipes. They are usually more predictable with regard to injured organs.

Nevertheless, a high index of suspicion must be maintained to avoid missing occult injuries [1]. Penetrating bladder injuries may be caused by injuries to the abdomen, thigh, or buttock just as rectal injuries. Any penetrating wound that may have injured the rectum should be fully evaluated to avoid severe complications [2].

This study aimed to report authors' experiences with associated bladder/rectal injuries in the last 22 years, bringing to light the importance of being aware of such injuries when treating a penetrating trauma.

## 2. Methods

This study represents the analysis of 2,693 trauma laparotomies in a level one trauma center after local Ethics Committee approval. A medical chart review from a prospectively maintained database was performed from January 1990 to December 2012 in the trauma surgery division of a university teaching hospital responsible for most of major traumatic and nontraumatic surgical emergencies in a metropolitan region consisting of 2.7 million inhabitants.

Attention to penetrating abdominal wounds was given and all penetrating bladder and rectal injuries were stratified aiming to determine injury frequency, bladder/rectal injury patterns, and complications.

Variables such as gender, age, penetrating mechanism (gunshot wound (GSW) or stab wound (SW)), systolic blood pressure (SBP), American Association for the Surgery of Trauma Organ Injury Scaling (AAST-OIS) [3], other associated injuries (i.e., ileum/jejunum, iliac vessels), injury severity score (ISS), urinary and nonurinary complications, and length of stay (LOS) were also analyzed. ISS is an anatomical scoring system, varying from 0 to 75, which provides an overall score for patients with multiple injuries. Scores from 16 to 25 represent severe anatomic injury and above 25 represent critical anatomic injury [4].

Bladder injuries were suspected when the mechanism of trauma was an evident cause of bladder injury or in the presence of macroscopic hematuria, being frequently diagnosed by retrograde plain film cystography and/or computed tomography (CT) cystography in cases of hemodynamically stable patients with acute abdominal pain and with no defined diagnosis yet [5]. The assuredness of the attending surgeon for a penetrating transperitoneal injury indicated surgical exploration with no diagnostic work- up. Eventually, missed intraoperative injuries were found. Rectal injuries were evaluated with digital examination, proctoscopy/sigmoidoscopy, and/or CT scan.

Treatment of the bladder was determined by the location and extent of injury was identified by the preoperative period. Briefly, intraperitoneal bladder ruptures were surgically repaired. Minor isolated extraperitoneal bladder injuries were managed nonoperatively with catheter drainage alone, prophylactic antibiotics, and a cystogram on the 10th to 14th day.

Major extraperitoneal ruptures in patients undergoing laparotomy for reasons other than urological injuries were repaired transvesically by opening the dome, avoiding violation of the pelvic hematoma. Intraperitoneal rectal injuries were treated either by primary suture, loop colostomy, and abdominal cavity drainage or, in cases of extensive traumatic injury, by Hartmann's procedure. Extraperitoneal rectal wounds were treated by either laminar or tubular drainage with or without primary suture. Interposition omental flap was systematically used between rectal and bladder injuries.

## 3. Results

**Figure 1:**
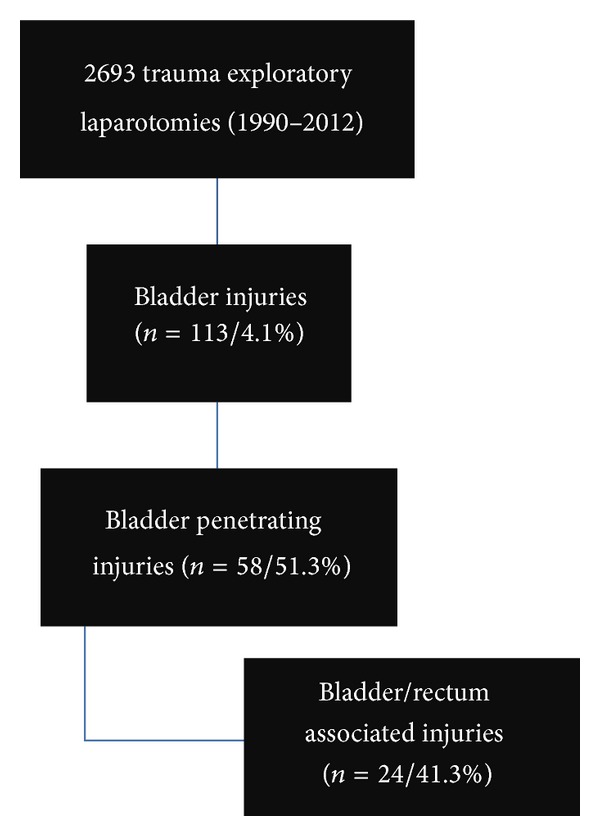
Study algorithm.

From the total 2,693 trauma laparotomies, 113 bladder lesions were found, representing 4.1% (113/2,693) rate. Penetrating injuries of the bladder were revealed to be the slight majority with 51.3% (*n* = 58) frequency, when compared with blunt trauma. From these, 41.3% (*n* = 24) were associated with rectal injuries (Figure 1). Males represented 95.8% of all associated bladder/rectal injuries, with a mean age of 29.8 years old.

GSW was the most common mechanism of injury to the bladder and rectum concomitantly (79.1%), followed by intentional or accidental impalement (20.9%). Regarding the clinical signs and symptoms, patients are most likely to arrive at the ER awake (91.6% with Glasgow coma scale 14-15), hemodynamically stable (systolic blood pressure average of 119.5 mmHg), with macroscopic hematuria (95.8%) and penetrating trauma stigmata (such as an impaled object or a GSW role) to the lower abdomen, buttock, thighs, or perineum (100%).

Physical exam appeared to be not sensitive for rectal injuries. Only 25% of the rectal injured patients presented with anal bleeding or blood on physical rectal exam. Three patients were later referred to our hospital (one GSW in buttock, one drop in iron bar, and other injured after falling in piece of wood) and operated between 6 and 12 hours after trauma. In only one patient with transfixing rectal injury, the posterior wall perforation was not identified during laparotomy and the patient developed pelvic abscess. The mean ISS was 19. A sum of 25% of injured patients arrived with an ISS above 25 despite “normal” systolic blood pressure.

Because of the evidence of abdominal cavity violation, most patients ended up immediately directed to the OR. Thus, the diagnostic work-up was not that often performed. Cystography was performed in 25% of the cases with a sensibility of 90%. CT scan was merely ordered in 12.5% of the cases. Intraperitoneal bladder injuries were more commonly present (60%).

Bladder surgical repair using absorbable suture was the elected treatment in all intraperitoneal injuries, associated lacerations, or extraperitoneal injuries in patients undergoing laparotomy for reasons other than urological injuries. The vast majority of extraperitoneal injuries were treated with a Foley catheter alone (87.6%), except those intraoperatively diagnosed, as mentioned above, which were surgically treated.

Rectal injuries AAST-OIS grade II (45.8%) were the most often lesions seen as associated with the bladder injury. Minimum stool spillage was found in 70.8% of all injuries. Sigmoid injuries were not frequently observed in our pool of patients (8.3%).

Additionally, rectal injuries were most likely to be intraperitoneal (66.6%) and AAST-OIS grade III and IV injuries were usually treated with primary suture plus protective colostomy (loop colostomy). In 6 patients, the treatment was primary repair without colostomy. When this was not possible due to a large defect of colon, Hartmann's procedure was performed, but this was infrequent (2 patients—8.3%). Extraperitoneal rectal injuries were insistently explored, primary suture was performed, and abdominal cavity was drained with a tubular or Jackson-Pratt drains.

Ten days was the average length of stay in the hospital. Complications were present in 20% of all operated patients; none of those resulted in death. Nonurinary (systemic/rectum) complications predominated (12%), including pneumonia, renal insufficiency, coagulopathy, sepsis, intra-abdominal abscess, and thromboembolic events. Urinary complications included urinary infections in seven patients (6.2%) and urinary fistula in two (1.8%).

## 4. Discussion

This study brings to light the importance of rectum-associated injuries in the presence of a bladder penetrating injury.

A nontreated or missed injury to the rectum can be devastating, evaluated with severe complications such as sepsis, rising up the morbidity of these traumatic injuries. With all that said, a high index of suspicion must be maintained to avoid missing occult injuries and treatment must be carried out as soon as possible. Penetrating trauma stigmata to the lower abdomen, thighs, or perineum is highly sensitive for bladder injuries, according to our series. In three patients evaluated in other facilities, the physicians did not suspect them to have rectal and bladder injuries because the patients were basically asymptomatic and there was a delay in sending them to our trauma center. A multidisciplinary approach involving general surgery is encouraged and in this context, urologists must be aware of common sites of injury during surgical exploration.

Furthermore, a heme-negative rectal examination should not preclude proctoscopy and eventually rectal surgical exploration, given that physical exam was not sensitive for rectal injuries with only 25% of the patients with rectal injuries presenting with anal bleeding or blood on physical and rectal exam. Harmonized with our previous study, with a high index of suspicion, concomitant bladder/rectum injuries are not a determinant prognosis factor once readily diagnosed and treated [6].

For patients who have penetrating trauma in the buttock area or lower abdomen, the lithotomy position may be required to approach any injury to the rectum. Particular attention must be paid to the retroperitoneal surfaces of the rectum; if necessary, the authors recommend mobilizing the right colon, opening the paracolic gutter to access the anatomic area.

Injuries to the extraperitoneal rectum are a distinct type of problem and require careful consideration of an assertive approach to management. They are different from colon or intraperitoneal rectal injuries because below the peritoneal reflection the rectum is encased by the mesorectum and surrounded by the soft tissues of the pelvis. It is often difficult to identify the site of injury at this level without full intraperitoneal mobilization of the rectum, which is not recommended by most authors, once there is no evidence that closure of extraperitoneal wounds is beneficial [7–10].

In hemodynamically stable patients, and when there is any doubt regarding abdominal cavity violation, CT scan becomes particularly useful, if it can determine a trajectory that is confirmatively outside the peritoneal cavity [11, 12]. Mandatory surgical intervention for penetrating abdominal trauma yields a high rate of negative laparotomies in the absence of visceral injuries. Laparoscopy is an alternative diagnostic procedure inspecting the peritoneum for signs of perforation and excluding significant intra-abdominal injuries [13–18].

Patients with any degree of hematuria after penetrating trauma must be carefully evaluated for kidney, ureteral, bladder, and urethral injuries. It is important to determine if bladder rupture is present and classify it as intraperitoneal (which requires exploration and repair) or extraperitoneal. Repair of extraperitoneal ruptures is indicated in patients undergoing laparotomy when careful inspection for associated lower urinary tract injuries is mandatory, and the surgeon can open the bladder at the dome and repair the injury from the inside [6].

Ureteral injury usually occurs after penetrating trauma. Direct inspection remains the fastest and most reliable method for detecting ureteric injury [17, 18]. An extended exploration of the retroperitoneum is mandatory in all cases of penetrating injury to this region. In cases of gunshot wounds, especially of high velocity, a meticulous exploration of the area of retroperitoneal violation must be done to avoid missing injuries secondary to the blast effect of missiles.

Even gross inspection may sometimes miss a blast effect, and there may be a role for postoperative intravenous urography in cases of high velocity gunshot wounds. Intravenous administration of either methylene blue or diuretics may identify the injury site when it is not obviously intraoperative [18].

In accordance with our numbers, GSW to the low urinary tract were recently identified in 50 patients of an American single center report, being 84% bladder injury (42 of 50), with a median age of 25 years and 94% of males; however, they found associated rectal injury in 34% (17 of 50) and higher likelihood of rectal injury with extraperitoneal bladder injury, whereas in the current study, rectal injuries occurred in 41.3% (24 patients) and were most likely to be intraperitoneal (66.6%) [19].

Confronting the literature, urinary fistula was seen in only two (1.8%) patients in this study, while Crispen et al. [20] noted fistula in 8% and 8% urinoma rates, and Franko et al. [21] reported rectovesical fistulas in 24% and abscess in 18%. These discrepancies may be rationalized by systematically omental flap interposition between rectal and bladder injuries in the current series.

While limited by regional and even cultural and developmental aspects of the study population, the current study adds to the expansion of urogenital trauma management, representing to the best of our knowledge one of the largest experiences reported in the literature of bladder/rectum associated injury.

Even restricted to descriptive and epidemiological aspects, the presented data shows that penetrating injuries to the bladder are highly associated with rectal injuries (41.3%) and with a high index of suspicion the relatively low rate of complications is in line with our previous study showing that concomitant rectum injuries were not a determinant prognosis factor [6]. Controversy persists regarding the management of penetrating rectal injuries, including injury repair, selective diversion, presacral drainage, and distal washout. Injuries to the proximal intraperitoneal and accessible distal one-third of the extraperitoneal rectum were treated with repair and selective colostomy.

Moreover, the current study methodological limitations are those inherent of trauma disease, given the unexpected and unpredictable way it occurs and is shared by most studies on the issue.

## 5. Conclusions

Bladder penetrating injuries are highly associated with rectal injuries (41.3%), sustaining a high index of suspicion to avoid missing occult injuries and late treatment. Rectal examination presents only 25% sensitivity and a heme-negative exam should not preclude proctoscopy and eventually rectal surgical exploration.
